# Vitamin D Supplementation and Recurrence of Benign Paroxysmal Positional Vertigo

**DOI:** 10.3390/nu16050689

**Published:** 2024-02-28

**Authors:** Guil Rhim, Moon-Jung Kim

**Affiliations:** 1 Department of Otorhinolaryngology, One Otorhinolaryngology Clinic, Paju 10924, Republic of Korea; guzi9170@hanmail.net; 2 Department of Laboratory Medicine, Konyang University College of Medicine, Daejeon 35365, Republic of Korea

**Keywords:** benign paroxysmal positional vertigo, vitamin D, recurrence, supplementation, treatment

## Abstract

Positional vertigo manifests as a spinning sensation triggered by changes in head position relative to gravity. Benign paroxysmal positional vertigo (BPPV) is an inner ear disorder characterized by recurrent episodes of positional vertigo. The connection between vitamin D insufficiency/deficiency and the onset and recurrence of BPPV is established. This study aims to assess vitamin D as a recurring factor in BPPV and the efficacy of vitamin D supplementation in preventing its recurrence. A comprehensive literature review on the relationship between vitamin D and BPPV recurrence was conducted, searching PubMed, Embase, Web of Science, and article reference lists for studies published from 2020 to 2023. A total of 79 articles were initially identified through the search, with 12 of them being utilized in the study. Recurrence rates for BPPV varied from 13.7% to 23% for studies with follow-up less than 1 year and 13.3% to 65% for studies with follow-up equal to or exceeding 2 years. Risk factors for BPPV recurrence include advanced age, female sex, hypertension, diabetes mellitus, hyperlipidemia, osteoporosis, and vitamin D deficiency. While earlier studies did not establish a link between low vitamin D levels and initial BPPV occurrence, they did associate recurrent episodes with low vitamin D levels. Recent research indicates that vitamin D supplementation in BPPV patients with deficiency or insufficiency decreases both the numbers of relapsing patients and relapses per patient. To validate these findings across diverse populations, further randomized controlled studies with larger cohorts and extended follow-up durations are essential.

## 1. Introduction

Vertigo is the perception of motion without actual movement, and positional vertigo involves a spinning sensation due to changes in head position relative to gravity. Benign paroxysmal positional vertigo (BPPV) is characterized by recurring episodes of positional vertigo.

Traditionally, the terms “benign” and “paroxysmal” categorize this specific form of positional vertigo, with “benign” historically implying that BPPV is not linked to any serious central nervous system disorder, suggesting a favorable prognosis for recovery [1].

Based on a neurotologic survey, vestibular vertigo’s lifetime prevalence and incidence were 7.8% and 1.5%, respectively [2]. One-year prevalence estimates were 4.9–5.2% for vertigo, 0.89% for migrainous vertigo, and 1.6% for benign paroxysmal positional vertigo. BPPV recurrence rates varied from 13.7% to 23% for studies with follow-ups less than 1 year and 13.3% to 65% for studies with follow-ups exceeding 2 years [3]. Overall, BPPV prevalence is reported as a lifetime prevalence of 2.4%, a 1-year prevalence of 1.6%, and a 1-year incidence of 0.6% [4,5]. Patients with BPPV often experience interruptions in their daily activities and may miss days at work, with studies indicating that up to 68% of individuals with BPPV reduce their workload, 4% opt to change jobs, and 6% ultimately resign due to the impact of the condition [6,7]. Furthermore, BPPV disproportionately affects older individuals, exacerbating its impact on health and quality of life, with older patients experiencing increased rates of falls, depression, and limitations in their daily activities. Persistent untreated or undiagnosed vertigo in the elderly leads to increased caregiver burden, resulting in societal costs such as an increased risk of nursing home placement and decreased family productivity [8].

The exploration of the correlation between vitamin D and BPPV commenced in the early 2010s. In a study conducted by Jeong et al., variations in 25(OH) vitamin D concentrations were observed when comparing 100 BPPV patients with a control group of 192 individuals, with values of 14.4 ± 8.4 ng/mL in the patient group and 19.0 ± 6.8 ng/mL in the control group, and vitamin D insufficiency (10–20 ng/mL), vitamin D deficiency (<10 ng/mL), and osteoporosis identified as significant factors influencing BPPV occurrence [9]. Buki et al. compared 14 patients without BPPV recurrence with four patients experiencing relapses, revealing a serum 25(OH) vitamin D concentration of 14 ng/mL in relapsing BPPV patients, lower than the concentration of 27 ng/mL in non-relapsing patients. Following an 8-month vitamin D supplementation therapy, no additional BPPV recurrence was observed in the treated patients [10].

Subsequent studies have revealed distinctions in serum vitamin D concentrations between recurrent and non-recurrent BPPV groups, with the recurrent group exhibiting lower levels of vitamin D [11,12].

Three meta-analyses examining the efficacy of vitamin D supplementation therapy for treating BPPV have been conducted based on articles published after 2020; however, the analyzed reference papers in these studies, comprising one randomized research study and six non-randomized research studies, all predate 2020 [13,14,15]. The conclusions of these three articles are consistent, indicating that vitamin D supplementation in BPPV patients with deficiency/insufficiency leads to lower recurrence rates compared to the control group. Nevertheless, the papers included in the meta-analyses exhibit considerable heterogeneity, which may impact their reliability and stability. In this study, we aimed to explore recent research findings on vitamin D supplementation therapy for BPPV after 2020 through a clinical review. While previous studies focused on differences in recurrence rates between control and intervention groups [16,17,18], recent research results demonstrate the efficacy of vitamin D supplementation in reducing recurrence frequency, particularly in patients with frequent recurrences of BPPV [19,20,21]. While previous studies did not find significant differences in recurrence rates between patients experiencing multiple recurrences and those with a single recurrence, recent research suggests that vitamin D supplementation has an effect on reducing the number of recurrences.

The review endeavors to assess the association between vitamin D and recurrent BPPV while examining the preventive benefits of vitamin D supplementation in recent research. By providing an overview of the current literature in this area of uncertainty, the aim is to contribute to understanding the efficacy of vitamin D supplementation in mitigating relapses among individuals experiencing high recurrence rates of BPPV.

## 2. Materials and Methods

**Figure 1:**
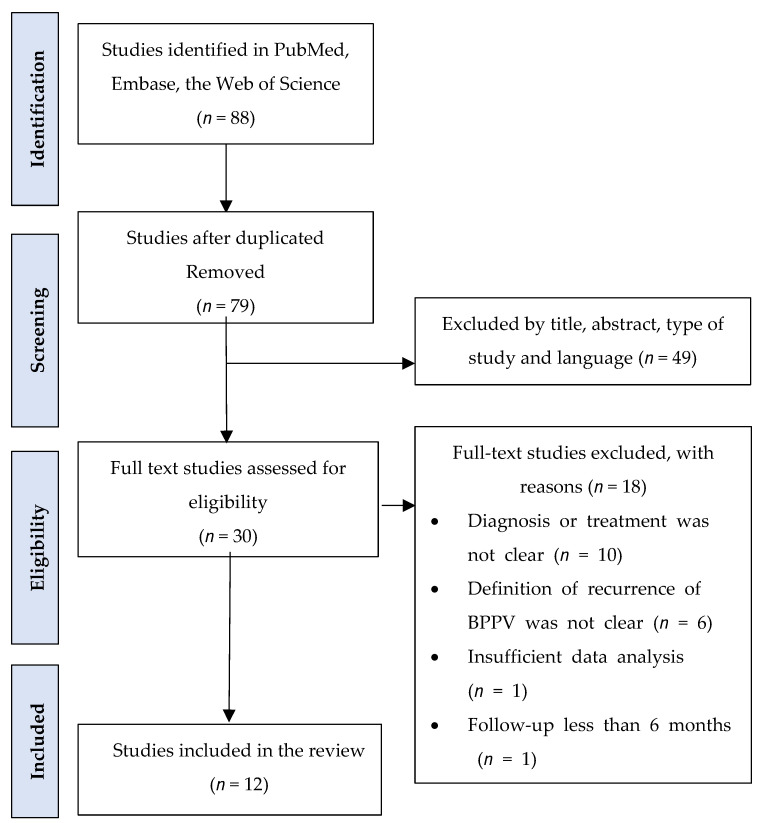
Flowchart of data search and studies selection.

A comprehensive literature review focusing on vitamin D and BPPV was conducted. Eligible studies were systematically identified through searches on PubMed, Embase, and the Web of Science, covering publications from 2020 to 2023 (Figure 1). The search criteria included terms such as “vitamin D”, “25(OH) vitamin D”, “25-hydroxyvitamin D”, in conjunction with “benign paroxysmal positional vertigo” or “BPPV”, and also incorporating terms related to “treatment” or “supplementation”. The search strategy yielded 79 articles. Initial screening of titles was performed by the corresponding author to determine eligibility, followed by independent abstract and full-text screening by all authors based on predetermined criteria.

The analysis included studies meeting the following eligibility criteria: retrospective or prospective investigations targeting the recurrence of BPPV and its correlation with vitamin D, BPPV diagnosis aligned with clinical practice guidelines, and articles published in English. Conversely, studies falling under the following exclusion criteria were omitted from the analysis: systematic reviews, case reports, experimental studies, studies lacking sufficient data for analysis, instances where the diagnosis, treatment, or definition of BPPV recurrence was unclear, and particularly in studies with follow-up durations less than 6 months or where it was left unstated.

Studies were initially identified based on their title and abstract, followed by a thorough assessment of eligibility through a full-text review to determine the final set of included studies. The extracted and evaluated data pertaining to the relationship between vitamin D and the recurrence of BPPV included details such as the first author, year of publication, mean age, population size, and vitamin D concentrations in cases and controls, as well as non-recurrent and recurrent instances.

Furthermore, for studies investigating the impact of vitamin D supplementation on the recurrence of BPPV, the extracted and evaluated data encompassed information such as the first author, year of publication, population size, follow-up periods, intervention details, vitamin D concentrations before and after treatment, and relevant outcomes.

## 3. Results

Eight articles were found to meet the eligibility criteria for the investigation of serum vitamin D levels and BPPV (Table 1).

Seven articles investigated serum vitamin D levels in both BPPV cases and controls. Among them, two articles reported a statistically significant difference, indicating that the mean serum vitamin D levels in the BPPV group were notably lower than those in the control group [24,27]. The reported serum vitamin D levels for BPPV cases and controls ranged from 17.1 to 31.4 ng/mL and 17.6 to 30.7 ng/mL, respectively, across the studies.

Additionally, three articles explored serum vitamin D levels in recurrent BPPV groups compared to non-recurrent BPPV groups. Two of these articles found that the recurrent BPPV group exhibited significantly lower serum vitamin D levels than the non-recurrent BPPV group [25,28].

We conducted a review of the study results on vitamin D supplementation for preventing recurrences of BPPV within the last 3 years (Table 2). Out of the five articles examined, the majority, four out of five, reported that vitamin D supplementation in BPPV patients effectively prevented recurrence of BPPV [19,20,21,29]. Consistently, these studies showed that the group supplemented with vitamin D exhibited a lower recurrence rate compared to the control group without vitamin D supplementation. The group receiving vitamin D supplementation showed a significant reduction in the frequency of recurrences [19]. Among these studies, two had a follow-up duration ranging from greater than 6 months to less than a year [16,26], while three had a follow-up time of 1 year or more [20,29,30].

The reported serum vitamin D levels for BPPV before and after supplementation were within the ranges of 6.06–20.18 ng/mL and 24.2–36.9 ng/mL, respectively, across the studies. Moreover, a significant difference in serum vitamin D levels was observed between the vitamin D supplementation group and the control group.

## 4. Discussion

Vitamin D, a lipid-soluble prehormone, plays a crucial role in maintaining calcium and phosphorus homeostasis. Vitamin D can be obtained through dietary intake, consumption of foods fortified with vitamin D, and skin exposure to ultraviolet B radiation. According to the US Institute of Medicine, serum 25(OH) vitamin D levels below 30 nmol/L (12 ng/mL) are considered deficient, levels between 30–50 nmol/L (12–20 ng/mL) are deemed insufficient, and levels equal to or above 50 nmol/L (20 ng/mL) are considered sufficient [31]. The Endocrine Society sets the threshold for deficiency at ≤50 nmol/L (20 ng/mL) and for insufficiency at 50–75 nmol/L (20–30 ng/mL) [32]. However, there is consensus across guidelines that serum 25(OH)D levels below 25 or 30 nmol/L (10–12 ng/mL) should be avoided at all ages [33]. The occurrence of vitamin D deficiency is elevated in regions situated at high latitudes, with winter–spring prevalence being 1.7 times that of summer–autumn, while the Eastern Mediterranean region and lower-middle-income countries also exhibit higher prevalence rates [34]. Females are more susceptible to vitamin D deficiency [31]. Heterogeneity among studies is attributed to factors such as season, sex, sampling region, detection assays, time of data collection, sampling frame, and other variables [33,34].

Significant variation exists in the prevalence of vitamin D deficiency across various WHO regions [34]. The Eastern Mediterranean region reports the highest prevalence, with 58.9% of the Kuwaiti population aged 10 years experiencing serum 25(OH)D levels of 12 ng/mL and 44.3% of Oman’s population aged 18–55 years facing similar levels. Despite abundant sunshine in the Middle East, cultural practices such as wearing veils contribute to limited sun exposure, explaining the high prevalence of vitamin D deficiency in these regions [35]. In the Americas, the prevalence is the lowest, with only 3.0% of the population aged 2 and older having serum 25(OH)D levels of 12 ng/mL, whereas in the European region, 18.0% and 53.0% of the population have serum 25(OH)D levels below 12 ng/mL and 20 ng/mL, respectively [34]. The Southeast Asian and Western Pacific regions also show a high prevalence of vitamin D deficiency, with 22.0% of the Southeast Asian population and 10.0% of the Western Pacific population having serum 25(OH)D levels below 12 ng/mL [35]. Notably, areas around 20–40° North latitude report the highest prevalence, with Saudi Arabia (37.4%), Bahrain (49.4%), and Iraq (31.1%) having a substantial portion of the population suffering from serum 25(OH)D levels below 12 ng/mL [34].

Studies on vitamin D and BPPV from the most recent 3 years were summarized for each continent (Table 1), revealing heterogeneity among the eight studies comparing vitamin D levels and conflicting results regarding the significant role of vitamin D deficiency in the underlying mechanism of BPPV. Mean serum vitamin D concentrations varied between institutions in the country of study.

Shin et al. conducted a study classifying patients into BPPV recurrence (Group A) and non-recurrence groups (Group B), showing a significant difference in average vitamin D levels between the two groups (12.9 ± 8.0 ng/mL for Group A and 19.2 ± 8.2 ng/mL for Group B; *p* = 0.011). However, no significant differences were observed in age, sex, cervical vestibular evoked myogenic potential, or ocular vestibular evoked myogenic potential between the two groups [28].

In line with these findings, another investigation indicated that individuals with recurrent BPPV exhibited notably diminished levels of vitamin D upon initial diagnosis, in contrast to those without recurrences (29.0 ± 12.0 vs. 37.6 ± 18.3 ng/mL; *p* = 0.012) [25]. Additionally, Resuli et al. reported that individuals in the BPPV group had significantly lower vitamin D levels than those in the control group [24]. Bazoni et al., on the other hand, found no association between 25(OH) vitamin D levels and BPPV in the general population, but noted an association with bone mineral density in the elderly group with diabetes mellitus and BPPV [22]. While some research findings suggested no association between vitamin D levels and serum calcium in BPPV, a negative correlation was identified between vitamin D concentrations and the frequency of BPPV episodes (*p* < 0.012) [23]. Several studies consistently reported low vitamin D status in patients with BPPV, with no significant differences in 25(OH)D levels between BPPV patients and controls [19,26].

The study by Sarsitthithum et al. found that mean serum vitamin D levels were lower in the BPPV group compared to the control group (*p* = 0.001). Additionally, within the BPPV participants, there was no statistically significant difference in mean serum vitamin D levels between those with recurrent BPPV and those with newly diagnosed BPPV (*p* = 0.313) [27]. Recent meta-analyses have indicated that BPPV is not associated with decreased levels of serum vitamin D, and low vitamin D levels are significantly evident among patients with recurrent episodes of BPPV [36,37].

In conclusion, while previous studies did not support the relationship between lower vitamin D levels and the occurrence of BPPV, they consistently support, with unremarkable heterogeneity, that recurrent episodes of BPPV have been associated with low levels of vitamin D.

Rhim reported on a study involving 99 patients with idiopathic BPPV and vitamin D deficiency, where 25 patients received 3 to 4 injections of 200,000 IU of vitamin D3 in the first year, while a control group consisting of 50 patients was selected through frequency matching, resulting in 25 patients in the case group. In terms of relapse rates between the entire case and control groups during different periods (0 to 6 months, 7 to 12 months, 13 to 24 months, and the entire study period), there were no statistically significant differences (*p* < 0.531, *p* < 1.000, *p* < 0.711, and *p* < 0.883, respectively), and the distribution of canalolithiasis and cupulolithiasis in BPPV types was 40 and 35, respectively [30].

Libonati et al. conducted a study reporting that oral nutritional supplementation with vitamin D3 plus antioxidants could prevent relapses in patients suffering from high-recurrence BPPV, assessing the BPPV recurrence rate based on patients experiencing two or more episodes of BPPV within the previous 6 months or at least three episodes in the previous 12 months [21]. A total of 128 patients with high-recurrence BPPV involving the posterior semicircular canal or the lateral semicircular canal (both in geotropic and apogeotropic forms) were randomized into three arms. After 6 months of follow-up, only Arm 1 exhibited a significant reduction in BPPV relapses compared to the baseline (−2.32, 95% confidence interval (CI) = −3.41–−1.62, *p* < 0.0001), with serum vitamin D levels of 18.2 ng/mL and 36.9 ng/mL for Arm 1 and Arm 2, respectively [21].

Pecci et al. conducted a study reporting that correcting hypovitaminosis (<30 ng/mL) could reduce both the number of patients experiencing relapses and the number of relapses per patient. Before integration, 100% of the patients had recurrent BPPV, whereas after supplementation, only 5 out of 16 patients (31.25%) had just 1 recurrence (*p* = 0.0003) [19].

Sanchez et al. conducted a study indicating a significant difference in the probability of recurrence between experimental groups, particularly Group 2, which received treatment involving repositioning maneuvers and vitamin D supplementation, showing a decreased recurrence of vertigo (*p* < 0.017), and the follow-up period ranged from 6 to 13 months [20].

Jeong et al. reported that considering calcium and vitamin D supplementation may be warranted for patients experiencing frequent BPPV episodes, particularly in cases of subnormal serum vitamin D levels (<20 ng/mL). They conducted an investigator-initiated, blinded-outcome assessor, parallel, multi-center, randomized controlled trial, wherein patients in the intervention group took vitamin D 400 IU and 500 mg of calcium carbonate twice a day for 1 year when serum vitamin D level was lower than 20 ng/mL, and patients in the observation group were assigned to follow-ups without further vitamin D evaluation or supplementation [29]. The intervention group showed a reduction in the annual recurrence rate (0.83 vs. 1.10). The proportion of patients with recurrence was lower in the intervention group than in the observation group (37.8 vs. 46.7%, *p* = 0.005).

The meta-analysis by Yang et al. revealed that in a randomized model, the group receiving vitamin D supplementation exhibited a lower recurrence rate compared to the control group without such supplementation (relative ratio = 0.41, 95% CI = 0.26–0.65; *p* < 0.01) [13]. Another meta-analysis also showed a significant preventive effect of vitamin D supplementation on BPPV recurrences (relative ratio = 0.37; 95% CI = 0.18–0.76; *p* = 0.007 with the random-effects model), and despite considerable heterogeneity among the studies, sensitivity analyses affirmed the reliability and stability of the results [14]. The study by Hong et al. reported that in a randomized clinical trial, vitamin D supplementation reduced the annual recurrence rate of vertigo in BPPV patients, while non-randomized clinical trials suggested a potential null effect in the random effects model (odds ratio = 0.08; 95% CI = 0.00–1.56) [15]. The randomized clinical trial was deemed to have a low risk of bias, while all nonrandomized studies were considered to have serious risks of bias. The existing literature emphasizes the role of optimizing vitamin D levels in BPPV patients, with consistent evidence from intervention studies showing a decrease in BPPV recurrence with vitamin D supplementation in individuals with below-normal vitamin D levels.

If there is already a systematic review on the same topic, sufficient quality assessment should be conducted to guide the direction of the new systematic review. Given the considerable differences in research design and methods, as well as the challenge of distinguishing between recurrence rates and non-recurrence rates and combining the intensity of recurrence frequency, it may be difficult. Even when using a random-effect model, the variability expressed as variability in results within-study and variability in results between-study diminishes the significance, making it difficult to analyze the combined effect estimates. Through the clinical reviews of the journals used in this study, it is evident that not only does the vitamin D supplementation group show differences in recurrence rates compared to the control group, as demonstrated in previous meta-analyses, but recent research consistently indicates a reduction in the frequency of recurrences, even in patients with vitamin D deficiency who undergo vitamin D supplementation.

The recurrence of BPPV may involve unidentified factors, and while vitamin D’s impact on otoconia is known through calcium regulation and ion channel/pump influence, its role in BPPV may extend beyond these mechanisms. Conditions like BPPV, acute hearing loss, idiopathic facial paralysis, and vestibular neuritis share etiological factors, suggesting a link to initial viral infection and subsequent autoimmune/autoinflammatory reactions, with some entities exhibiting varying degrees of demyelination [38]. Vitamin D’s immunomodulatory impact could potentially curb post-viral autoimmune responses, bolstered by its antioxidative properties and its potential to stabilize endothelial cells. The correlation between insufficient vitamin D levels and demyelination, established in conditions such as multiple sclerosis, implies a comparable involvement in BPPV, with research on mice deficient in vitamin D receptors unveiling degenerative alterations in the inner ear ganglia, hair cells, and otoconia [39].

Nakada et al. elucidated the difference in serum vitamin D levels between canalolithiasis and cupulolithiasis of the horizontal canal, with patients diagnosed with canalolithiasis showing a mean 25(OH)D serum level of 13.2 ± 1.4 ng/mL and those with cupulolithiasis exhibiting 20.4 ± 1.6 ng/mL, demonstrating a statistically significant disparity (*p* = 0.0014) [40]. These findings indicate pathophysiological distinctions in vitamin D concerning canalolithiasis and cupulolithiasis, offering insights into the results of Rhim’s study. Familial socioeconomic factors, influencing diet quality, reduced oily fish intake, supplement use, and limited access to outdoor amenities, may impact vitamin D levels [41]. Recent studies on metabolic diseases and BPPV suggest increased recurrences with more chronic diseases, like diabetes and hypertension, related to socioeconomic status. Vitamin D supplementation therapy might be effective under specific conditions.

This review aims to examine the research outcomes of vitamin D supplementation in BPPV after 2020 and confirm similar conclusions to those of studies conducted before 2020. Additionally, it was observed that vitamin D supplementation can significantly reduce the frequency of recurrences even in severe relapsing BPPV patients, thus improving their quality of life. However, among the studies included in the clinical review, there was only one paper that reported no efficacy of vitamin D supplementation, and the analysis of why there was no effect was lacking. Results that are not statistically significant may face challenges in being published. Furthermore, to date, there have been more observational studies describing significant effects of vitamin D supplementation than randomized controlled trials. This characteristic may lead to an overestimation of the analysis results in meta-analyses combining published research outcomes with reality.

## 5. Conclusions

Recent research indicates that vitamin D supplementation in BPPV patients with deficiency or insufficiency reduces both the number of relapsing patients and relapses per patient. It is advisable for clinicians to investigate and address vitamin D deficiency in individuals with BPPV. However, a substantial number of studies employed non-randomized designs, and information on control groups was frequently insufficient. Consequently, additional randomized controlled studies are essential to validate these research findings. These studies should involve larger cohorts of patients and implement extended follow-up periods to ensure validation across populations with diverse medical, economic, and social backgrounds.

## Figures and Tables

**Table 1:** Main characteristics of recent studies on benign paroxysmal positional vertigo and serum vitamin D.

Author (Year)	Country	Age (Year)	Case/ Control	Vit. D in Case ^a^	Vit. D in Control ^a^	Vit. D in Non-Re Current ^a^	Vit. D in Recurrent ^a^	*p*-Value
Bazoni (2020) [22]	Brazil	68.7	17/92	27.8	23.8	NA	NA	0.260
Thomas (2021) [23]	India	44.4	49/49	21.3	17.6	NA	NA	0.243
Resuli (2022) [24]	Turkey	44	258/100	18.8	30.7	NA	NA	0.000
Pecci (2022) [19]	Italy	62	26/24	20.2	23.7	NA	NA	0.160
Cobb (2023) [25]	USA	66.2	173/5962	31.4	26.0	37.6	29.0	0.012 ^b^
Ren (2023) [26]	China	60	182/182	17.1	19.2	NA	NA	0.328
Sarsitthithum (2023) [27]	Thailand	60.5	69/68	21.5	26.3	21.0	21.9	0.313 ^b^
Shin (2023) [28]	Korea	48	332/NA	14.7	NA	12.9	19.2	0.001 ^b^

NA, not available; ^a^, ng/mL; ^b^, statistical difference between non-recurrent and recurrent.

**Table 2:** Effects of vitamin D supplementation observed in this review.

Author (Year)	Population	Intervention	Case/ Control	Follow Up (Months)	Vitamin D before Treatment ^a^	Vitamin D after Treatment ^a^	Outcomes
Rhim (2020) [30]	BPPV with vitamin D lower than 10 ng/mL	3 to 4 injections totaling 200,000 IU of vitamin D3 within the first year	24/50	24	6.06	31.1	No significant differences
Jeong (2020) [29]	BPPV with vitamin D lower than 20 ng/mL	vitamin D 400 IU and 500 mg of calcium carbonate twice a day for 1 year	448/512	12	13.3	24.2	Proportion of patients with recurrence was lower in the intervention than in the observation group (37.8 vs. 46.7%, *p* = 0.005)
Sánchez (2022) [20]	BPPV with vitamin D lower than 30 ng/mL	oral cholecalciferol 16,000 UI once a week during 8–10 weeks	35	6–13	18.5	26.2	Intervention group had a decreased recurrence of vertigo (*p* = 0.017)
Pecci (2022) [19]	BPPV with vitamin D lower than 30 ng/mL	oral cholecalciferol 25,000–50,000 IU once a week for 4 weeks, then 7000 IU once a week for 2 months	26/24	4–8	20.18	28.1	5/16 patients (31.25%) had only 1 recurrence (average number of relapses/patient: 0.31, *p* = 0.0003)
Libonati (2022) [21]	BPPV with vitamin D lower than 30 ng/mL	vitamin D3 800 IU daily for 6 months	128	6	18.2	36.9	A significant reduction in BPPV relapses compared to the baseline was observed (*p* < 0.0001)

^a^, ng/mL.

## References

[1] Baloh R.W., Honrubia V., Jacobson K. (1987). Benign positional vertigo: Clinical and oculographic features in 240 cases. Neurology.

[2] Neuhauser H.K. (2007). Epidemiology of vertigo. Curr. Opin. Neurol..

[3] Neuhauser H.K., von Brevern M., Radtke A. (2005). Epidemiology of vestibular vertigo: A neurotologic survey of the general population. Neurology.

[4] von Brevern M., Radtke A., Lezius F. (2007). Epidemiology of benign paroxysmal positional vertigo: A population based study. J. Neurol. Neurosurg. Psychiatry.

[5] Sfakianaki I., Binos P., Karkos P. (2021). Risk factors for recurrence of benign paroxysmal positional vertigo. a clinical review. J. Clin. Med..

[6] Li J.C., Li C.J., Epley J. (2000). Cost-effective management of benign positional vertigo using canalith repositioning. Otolaryngol. Head Neck Surg..

[7] Benecke H., Agus S., Kuessner D. (2013). The burden and impact of vertigo: Findings from the REVERT patient registry. Front. Neurol..

[8] Bhattacharyya N., Gubbels S.P., Schwartz S.R. (2017). Clinical practice guideline: Benign paroxysmal positional vertigo (Update). Otolaryngol. Head Neck Surg..

[9] Jeong S.H., Kim J.S., Shin J.W. (2013). Decreased serum vitamin D in idiopathic benign paroxysmal positional vertigo. J. Neurol..

[10] Bűki B., Ecker M., Jűnger H. (2013). Vitamin D deficiency and benign paroxysmal positioning vertigo. Med. Hypotheses.

[11] Talaat H.S., Abuhadied G., Talaat A.S. (2015). Low bone mineral density and vitamin D deficiency in patients with benign positional paroxysmal vertigo. Eur. Arch. Otorhinolaryngol..

[12] Rhim G.I. (2016). Serum vitamin D and recurrent benign paroxysmal positional vertigo. Laryngoscope Investig. Otolaryngol..

[13] Yang Z., Li J., Zhu Z. (2021). Effect of vitamin D supplementation on benign paroxysmal positional vertigo recurrence: A meta-analysis. Sci. Prog..

[14] Jeong S.H., Lee S.U., Kim J.S. (2022). Prevention of recurrent benign paroxysmal positional vertigo with vitamin D supplementation: A meta-analysis. J. Neurol..

[15] Hong X., Christ-Franco M., Moher D. (2022). Vitamin D supplementation for benign paroxysmal positional vertigo: A systematic review. Otol. Neurotol..

[16] Talaat H.S., Kabel A.-M.H., Khaliel L.H. (2016). Reduction of recurrence rate of benign paroxysmal positional vertigo by treatment of severe vitamin D deficiency. Auris Nasus Larynx.

[17] Sheikhzadeh M., Lotfi Y., Mousavi A. (2016). The effect of serum vitamin D normalization in preventing recurrences of benign paroxysmal positional vertigo: A case-control study. Casp. J. Intern. Med..

[18] Califano L., Salafa F., Melillo M.G. (2019). Is hypovitaminosis D a risk factor for either the onset or the recurrence of benign paroxysmal positional vertigo?. Front. ORL.

[19] Pecci R., Mandalà M., Marcari A. (2022). Vitamin D insufficiency/deficiency in patients with recurrent benign paroxysmal positional vertigo. J. Int. Adv. Otol..

[20] Sánchez J.M., Leonardo J.C.H., Niembro J.K.I. (2022). Therapeutic effect of the correction of vitamin D deficiency in patients with benign paroxysmal positional vertigo. a randomized clinical trial. Int. Arch. Otorhinolaryngol..

[21] Libonati G.A., Leone A., Martellucci S. (2022). Prevention of recurrent benign paroxysmal positional vertigo: The role of combined supplementation with vitamin D and antioxidants. Audiol. Res..

[22] Bazoni J.A., Ciquinato D.S., Marquez A.d.S. (2020). Hypovitaminosis D, low bone mineral density, and diabetes mellitus as probable risk factors for benign paroxysmal positional vertigo in the elderly. Int. Arch. Otorhinolaryngol..

[23] Thomas R.J., Goutham M.K., Bhat V.S. (2022). Association of serum calcium and vitamin D with benign paroxysmal positional vertigo. Int. Arch. Otorhinolaryngol..

[24] Resuli A.S., Bedir A., Özgür A. (2022). The relationship between benign paroxysmal positional vertigo and vitamin D. Cureus.

[25] Cobb L.H., Bailey V.O., Liu Y.F. (2023). Relationship of vitamin D levels with clinical presentation and recurrence of BPPV in a Southeastern United States institution. Auris Nasus Larynx.

[26] Ren Y.Y., Wang Y.J., Li J.L. (2023). Low vitamin D and uric acid status in patients with benign paroxysmal positional vertigo. Sci. Prog..

[27] Sarsitthithum K., Wisupagan T., Kiatthanabumrung S. (2023). The association between serum vitamin D levels and benign paroxysmal positional vertigo. Ear Nose Throat J..

[28] Shin H.I., Park Y., Lee H.J. (2023). Correlation between serum vitamin D level and benign paroxysmal positional vertigo recurrence. Auris Nasus Larynx.

[29] Jeong S.H., Kim J.S., Kim H.J. (2020). Prevention of benign paroxysmal positional vertigo with vitamin D supplementation. Neurology.

[30] Rhim G.I. (2020). Effect of vitamin D injection in recurrent benign paroxysmal positional vertigo with vitamin D deficiency. Int. Arch. Otorhinolaryngol..

[31] Ross A.C., Manson J.E., Abrams S.A. (2011). The 2011 report on dietary reference intakes for calcium and vitamin D from the Institute of Medicine: What clinicians need to know. J. Clin. Endocrinol. Metab..

[32] Valcour A., Blocki F., Hawkins D.M. (2012). Effects of age and serum 25-OH-vitamin D on serum parathyroid hormone levels. J. Clin. Endocrinol. Metab..

[33] Bouillon R. (2017). Comparative analysis of nutritional guidelines for vitamin D. Nat. Rev. Endocrinol..

[34] Cui A., Zhang T., Xiao P. (2023). Global and regional prevalence of vitamin D deficiency in population-based studies from 2000 to 2022: A pooled analysis of 7.9 million participants. Front. Nutr..

[35] Hilger J., Friedel A., Herr R. (2014). A systematic review of vitamin D status in populations worldwide. Br. J. Nutr..

[36] AlGarni M.A., Mirza A.A., Althobaiti A.A. (2018). Association of benign paroxysmal positional vertigo with vitamin D deficiency: A systematic review and meta-analysis. Eur. Arch. Otorhinolaryngol..

[37] Yang B., Lu Y., Xing D. (2020). Association between serum vitamin D levels and benign paroxysmal positional vertigo: A systematic review and meta-analysis of observational studies. Eur. Arch. Otorhinolaryngol..

[38] Goldschagg N., Teupser D., Feil K. (2021). No evidence for a specific vitamin D deficit in benign paroxysmal positional vertigo. Eur. J. Neurol..

[39] Büki B., Jünger H., Zhang Y. (2019). The price of immune responses and the role of vitamin D in the inner ear. Otol. Neurotol..

[40] Nakada T., Sugiura S., Uchida Y. (2019). Difference in serum levels of vitamin D between canalolithiasis and cupulolithiasis of the horizontal semicircular canal in benign paroxysmal positional vertigo. Front. Neurol..

[41] Roberts K., Cade J., Dawson J. (2018). Empirically derived dietary patterns in UK adults are associated with sociodemographic characteristics, lifestyle, and diet quality. Nutrients.

